# Intussusception following rotavirus vaccination: a reminder for practitioners

**DOI:** 10.3399/bjgpopen17X100629

**Published:** 2017-01-09

**Authors:** Vijay Chavda, Lucy Henderson, Rishi Chavda, Suchandra Pande, Khalid ElMalik

**Affiliations:** 1 Core Surgical Trainee, East Midlands South Deanery, Department of Paediatric Surgery, Leicester Royal Infirmary, Leicester, UK; 2 Speciality Trainee in Paediatric Surgery, Department of Paediatric Surgery, Leicester Royal Infirmary, Leicester, UK; 3 Foundation Year 1 Doctor, East Midlands North Deanery, Department of Anaesthetics and Intensive Care, Lincoln County Hospital, Lincoln, UK; 4 Consultant Paediatrician and Gastroenterologist, Department of Paediatrics, Leicester Royal Infirmary, Leicester, UK; 5 Consultant in General Paediatric Surgery and Oncology, Department of Paediatric Surgery, Leicester Royal Infirmary, Leicester, UK

**Keywords:** interssusception, rotavirus, vaccination, paediatric surgery

## Introduction

Intussusception is a paediatric surgical emergency that requires prompt diagnosis and management to prevent significant patient morbidity and mortality. Of the recognised causes, intussusception following rotavirus vaccination is relatively rare and may be overlooked by practitioners. We present the case of a 2-month-old infant found to have intussusception following rotavirus vaccination together with a review of current literature around this important topic.

## Case report

A 2-month-old male infant was admitted under the paediatric team with a 1-day history of non-bilious vomiting, pyrexia, and irritability a day after receiving his first-dose rotavirus vaccination. On examination he was haemodynamically stable and had no focal signs of sepsis. His abdominal examination revealed a soft, non-distended abdomen with no palpable masses. He had passed normal stool within the preceding 24 hours. Following initial assessment, he underwent a full septic screen including lumbar puncture, the results of which were all within normal range.

**Figure 1.**
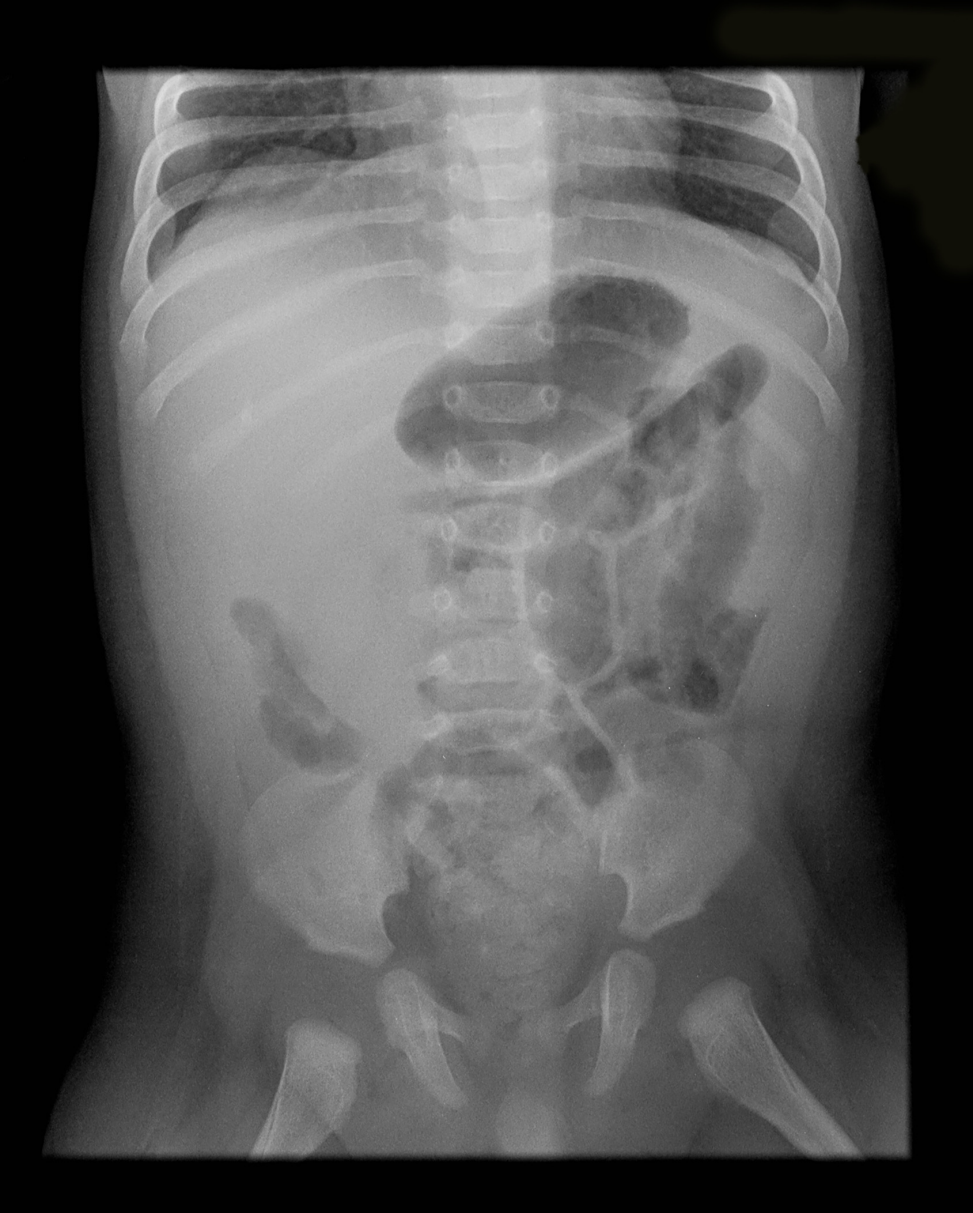
Abdominal X-ray showing small bowel obstruction.

**Figure 2.**
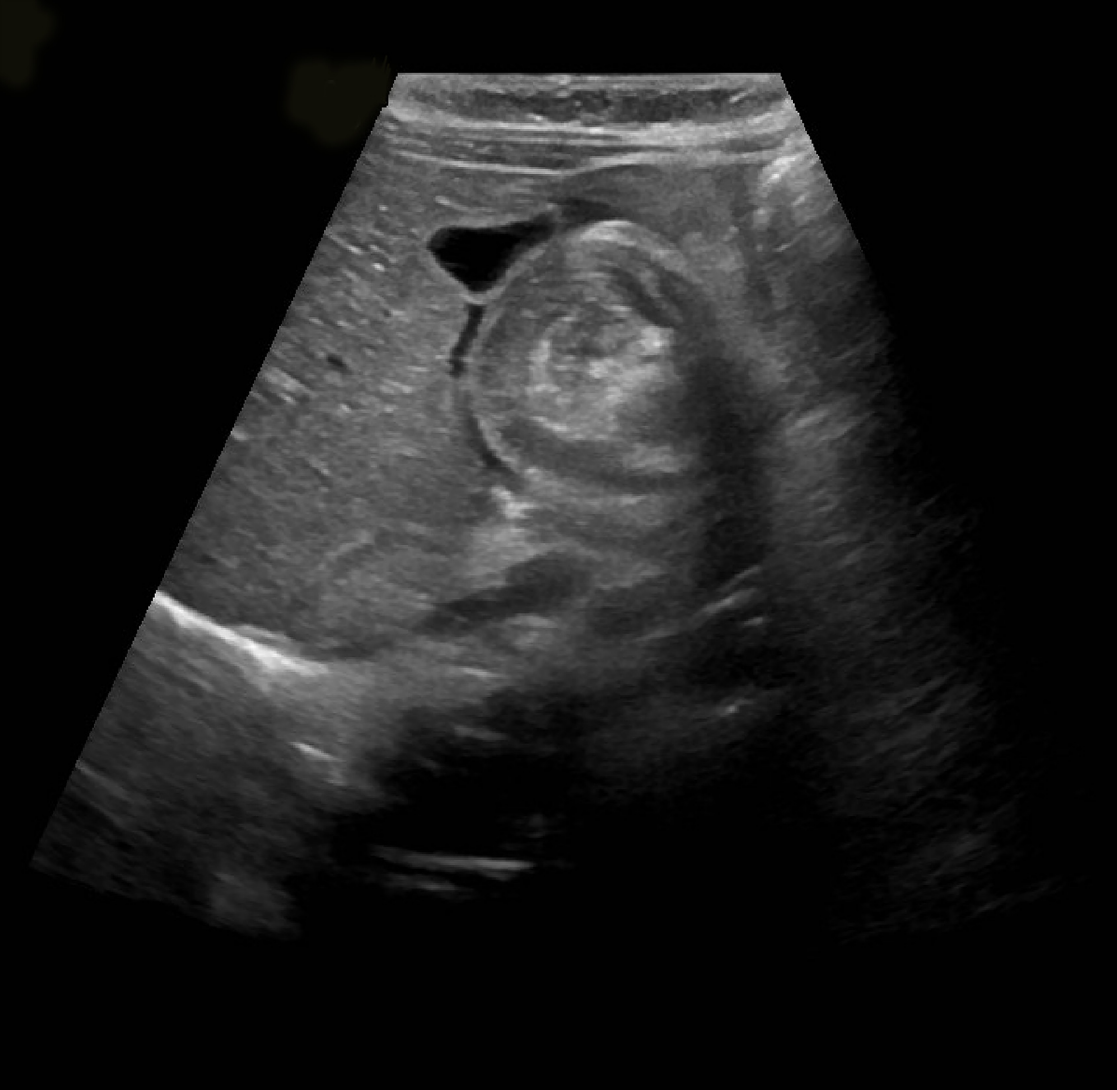
Ultrasound image showing characteristic target sign.

After developing bilious vomiting overnight a paediatric surgical review was obtained and an upper gastrointestinal contrast study was performed. This revealed no evidence of malrotation. An abdominal X-ray was subsequently performed which revealed a soft tissue mass in the right hypochondrium, dilated proximal small bowel loops, and a paucity of distal bowel gas, in keeping with small bowel obstruction (Figure 1). An urgent ultrasound scan was obtained which showed dilated proximal small bowel loops and the characteristic target sign typically seen in intussusception (Figure 2). The child received full resuscitation before an air enema reduction was performed under fluoroscopic guidance. This was successful at first attempt.

The following day the patient was well, tolerating feeds, and passing normal stools. He was subsequently discharged home. Following discussion with Public Health England the child’s parents were advised to decline the second-dose rotavirus vaccination.

## Background

### The rotavirus vaccine

The rotavirus vaccination programme in the UK consists of the oral administration of a live attenuated vaccine Rotatrix (GlaxoSmithKline, UK). Infants are typically given two doses of the vaccine; the first dose at 6–8 weeks and the second dose 4 weeks later. In a large-scale randomised controlled trial vaccination reduced the incidence of severe rotavirus-associated gastroenteritis and hospitalisation by 84.7% and 85.0% respectively.^1^

Prior to the introduction of the Rotatrix vaccine, its predecessor RotaShield (Wyeth Pharmaceuticals, US) was withdrawn from clinical use in 1999 due to a significant number of vaccinated patients developing intussusception; the highest risk being demonstrated 3–7 days after receiving the first dose (adjusted odds ratio 37.2, 95% confidence interval [CI] = 12.6 to 110.1). This translated to an additional 10.6–21.4 cases of intussusception per 100 000 infants vaccinated.^2^

Following introduction of the Rotatrix vaccine in 2008, a large-scale prospective surveillance programme conducted in Mexico examined the incidence of intussusception following vaccination. There was a statistically significant association for the development of intussusception within 7 days of receiving the first dose (relative incidence 6.49, 95% CI = 4.17 to 10.09, *P*<0.001); and the second dose of the vaccine, relative incidence 1.29 (95% CI = 0.80 to 2.11, *P *= 0.29). This equated to an additional 3–4 cases per 100 000 vaccinated infants.^3^

### Intussusception

Intussusception occurs when a proximal segment of bowel, along with its mesentery, invaginates into a distal segment of bowel. Mesenteric compression leads to bowel wall oedema and obstruction. Left untreated, there can be progression to ischaemia, infarction, perforation, and faecal peritonitis with a significantly increased morbidity and mortality, as well as a prolonged inpatient stay. In a retrospective study of 98 patients with intussusception, there was a higher incidence of open surgery in those patients that had symptoms for 24 hours or longer.^4^

A prospective surveillance study conducted in 2008–2009 found the incidence of intussusception in the UK to be 24.8 cases per 100 000 live births with the highest incidence found in the fifth month of life.^5^

The causes of intussusception can be divided into those with a pathological lead point, in which there is an abnormal pathology of the bowel acting as a focus for the intussusception (10% of cases), and those without (90%). The presence of a pathological lead point is more likely in children presenting at an older age. The main causes of intussusception are listed in Box 1.

**Box 1.** Main causes of intussusception

**Pathological leadpoint**	**Non-pathological leadpoint**
**Meckel’s diverticulum**	**Viral illness (typically rotavirus and adenovirus)**
Intestinal polyposis	Peyer’s Patch hypertrophy
**Peutz-Jeghers Syndrome**	**Idiopathic**
Lymphoma	
**Henoch-Schönlein purpura**	

The common presenting features of a child with intussusception are listed in Box 2. Although the symptoms and signs listed are felt to be typical of intussusception there are many pathologies that can present in a similar manner and, more importantly in a paediatric population, there may be not be classical signs and symptoms demonstrated, therefore a high index of suspicion is crucial.

**Box 2.** Key features of intussusception

Intermittent, paroxysmal episodes of severe colicky abdominal pain (in early disease the child may appear well in between episodes). In infant populations episodes may be observed as a distressed inconsolable child and there may be drawing up of the legs. Vomiting which is often bilious. Mucus per rectum or the typical ‘redcurrant jelly stools’. Peritonism, signs of sepsis, and shock are features of late disease and are suggestive of perforation.

In early disease symptoms may be non-specific, such as intermittent episodes of abdominal pain, between which the child may appear entirely well. Examination during early disease may also be unremarkable. The initial presentation of a child with early disease is often to GPs and, when faced with a child with vague symptoms and a normal examination, it is comprehensible that some children with early intussusception may be missed. The likelihood of surgical intervention increases with delayed diagnosis, together with a higher morbidity and mortality. Therefore, when faced with a child with non-specific symptoms and a recent history of rotavirus vaccination, early referral for further assessment in the secondary care setting is advised. If children are managed expectantly in the community or are discharged from hospital following review, their carers should be fully informed of ‘red flag’ symptoms and there should be relevant safety netting in place, such as open access to secondary care triage services, to allow rapid reassessment should a child’s symptoms fail to resolve or worsen.

Ultrasonography is the gold-standard investigation for suspected intussusception with the characteristic target sign being pathognomonic. In the absence of peritonitis reduction can be attempted with either a contrast enema or, more commonly, with an air enema. Indications for open surgery include signs of peritonism, radiological evidence of perforation, haemodynamic compromise, sepsis, or the presence of a pathological lead point.^6,7^

### Conclusion

The association between intussusception and rotavirus vaccination has been well documented and validated in the literature. The small increased risk of intussusception following vaccination with the Rotatrix vaccine is far outweighed by the large scale benefits of protection against severe rotavirus induced gastroenteritis in the paediatric population, a position which is upheld by the World Health Organization.^8^

The value of the rotavirus vaccine in conferring protection to vulnerable infants is perhaps best appreciated in developing countries where access to life saving emergency medical attention following severe gastroenteritis may not be as readily accessible in comparison to westernised populations.

This case reiterates the association between intussusception and rotavirus vaccination and we recommend that awareness of this uncommon but significant association should lead to a lower threshold for investigation when a recent of history of rotavirus vaccination is present.
